# Post-emergence herbicidal activity of nanoatrazine against *Alternanthera tenella* Colla plants compared to other weed species^☆^

**DOI:** 10.1016/j.heliyon.2022.e09902

**Published:** 2022-07-08

**Authors:** Bruno Teixeira de Sousa, Anderson do Espirito Santo Pereira, Leonardo Fernandes Fraceto, Halley Caixeta Oliveira, Giliardi Dalazen

**Affiliations:** a Department of Agronomy, Londrina State University (UEL), PR 445, km 380, 86057-970 Londrina, PR, Brazil; b Institute of Science and Technology, São Paulo State University (UNESP), Av. Três de Março 511, 18087-180 Sorocaba, SP, Brazil; c Department of Animal and Plant Biology, Londrina State University (UEL), PR 445, km 380, 86057-970 Londrina, PR, Brazil

**Keywords:** Atrazine, Calico plant, Meta-analysis, Nanoherbicides, Weed control

## Abstract

The encapsulation of atrazine into poly(epsilon-caprolactone) nanocapsules has been shown to improve the efficiency of the herbicide and decrease its environmental impacts. In the current work, we evaluated the efficiency of nanoatrazine in the post-emergence control of *Alternanthera tenella* Colla plants and performed a meta-analysis to compare the results with studies already published with other weeds. The first experiment was carried out in the field, where we observed that nanoatrazine (at 200 g a. i. ha^−1^) induced higher inhibition of the maximum quantum efficiency of photosystem II (up to 39%) than conventional atrazine at the same concentration. However, nanoencapsulation did not improve the visually-determined weed control by atrazine. To better understand the response of *A. tenella* plants to nanoatrazine, a second experiment was carried out in a greenhouse with four-leaf stage plants treated with nano and conventional atrazine at 200, 500, 1000, and 2000 g a. i. ha^−1^. Nanoatrazine showed higher efficiency (up to 33%) than commercial atrazine in inhibiting photosystem II activity at all doses until 48 h after application. Again, weed control and plant dry mass did not differ between formulations. From the meta-analysis, it was observed that *A. tenella* plants showed a response to nanoatrazine that differs from other target species, as the gain in efficiency resulting from the nanoencapsulation was restricted to the short-term analysis, and did not result in better weed control. These results reinforce that the efficiency of nanoatrazine is dependent on the studied species.

## 1 Introduction

Nanotechnology has many potential uses in agriculture, such as the development of nano-enabled pesticides. These formulations are developed from active ingredients available on the market, with the principal objective of reducing their negative effects on the environment (Pereira et al., 2021). In addition, new features are added, such as improved efficiency, stability, and solubility of the formulation (Kah, 2015; Pascoli et al., 2018). The active ingredient atrazine belongs to group of the triazinic herbicides [photosystem II (PSII) inhibitors], which is recommended for the pre- and post-emergence control of dicotyledonous plants and some grass weeds in maize, sugarcane and sorghum fields (Hansen et al., 2013; Recker et al., 2015; Shaner, 2014). Although it is a herbicide of great agronomic importance, the use of atrazine on a large scale is associated with contamination of surface and groundwater and, in addition to its toxicity to non-target organisms and high persistence in the environment (41–231 days), it was banned in several European countries (Hansen et al., 2013; Singh et al., 2018). For these reasons, it is necessary to optimize the use of atrazine by reducing its application dose and environmental impacts.

The nanoformulation of atrazine (nanoatrazine), produced by the nanoencapsulation of the active ingredient using the polymer poly(epsilon-caprolactone) (PCL), showed a reduction in the negative effects of the herbicide on non-target species and better weed control than a commercial, non-nano atrazine formulation, thus providing agronomic benefits (Grillo et al., 2012; Pereira et al., 2014; Oliveira et al., 2015; Sousa et al., 2018, 2020; Preisler et al., 2020; Zhai et al., 2020; Takeshita et al., 2021). In post-emergence, the higher efficiency of nanoatrazine has been related to improved uptake and distribution of the herbicide in the leaves, as nanoencapsulation provided a better interaction with plant tissues, allowing uptake by the stomatal pathway and greater mobility in vascular tissues (Bombo et al., 2019; Takeshita et al., 2021). Due to the rapid action of nanoatrazine, it is necessary to utilize sensors that monitor the herbicidal activity before the appearance of macroscopic symptoms. The measurement of the quantum maximum efficiency of PSII (F_v_/F_m_) using a portable fluorometer is an alternative to monitor the inhibitory effects of atrazine (Girotto et al., 2010).

For the post-emergent control of *Brassica juncea* (L.) Czern, *Amaranthus viridis* L., and *Bidens pilosa* L., nanoencapsulation provided expressive gains in the efficiency of the herbicide, so that a 10-fold reduction in nanoatrazine dosage yielded the same PSII inhibition and symptom evolution as commercial atrazine at 2000 g active ingredient (a. i.) ha^−1^ (Oliveira et al., 2015; Sousa et al., 2018). In *Digitaria insularis* (L.) Fedde, an atrazine tolerant species, and *Raphanus raphanistrum* L., a susceptible species, the application of nanoatrazine resulted in values for the inhibition of PSII activity up to 50% and 70% higher than the inhibition induced by commercial atrazine, respectively (Sousa et al., 2020; Takeshita et al., 2021). The post-emergence control of these two weed species was similar between 1000 g a. i. of nanoatrazine and 2000 g a. i. of commercial atrazine. Thus, for the species studied to date, atrazine nanoencapsulation enabled reductions of between 50 and 90% in the applied dose of the herbicide, without compromising its action on target plants. Furthermore, nanoatrazine led to higher impacts on protein content and oxidative stress parameters of *Lactuca sativa* L. plants compared to commercial atrazine, and the formulations differed in their effects on nutrient levels (Wu et al., 2021).

Aimed at agricultural applications, nanoencapsulation of herbicides represents a strategy to decrease the amount of the active ingredient released in the environment, reducing its environmental impacts and, hence, making its application safer. Recently, it has been suggested that atrazine degradation by rhizophere bacterial communities is promoted when the nanoencapsulated herbicide is applied (Zhai et al., 2020).

However, the number of weed species evaluated for the herbicide action of nanoatrazine is still limited. Thus, novel studies with the application of nanoatrazine to other weed species need to be carried out, in order to ratify its efficiency. *Alternanthera tenella* Colla (Amaranthaceae) is an herbaceous weed species native to the neotropics, that is able to form a wide cover on the soil due to intense branching (Canossa et al., 2007; Sánchez-del Pino and Iamonico, 2016). It is an annual weed with an erect stem (glabrous or slightly pubescent), dark-green sessile leaves (ovate or lanceolate), acute apex, and inflorescence arranged in globose glomeruli (Iamonico and Sánchez-del Pino, 2016). The leaves of *A. tenella* have a thick cuticle and a layer of wax deposition, in addition to low stomatal density on both sides, which can compromise the action of herbicides (Ferreira et al., 2003). It usually occurs in pastures and annual crop fields, where it may appear late, hindering the harvest and increasing the grain moisture (Canossa et al., 2007; Cardoso et al., 2017). Although there are no reports about herbicide resistance of *A. tenella* populations, it remains a problem due to its potential to produce an enormous number of seeds (Kissmann and Groth 1999).

This weed species has significant importance in agricultural areas with the cultivation of maize (one of the cultures for which atrazine is recommended) and cowpea (Alcântara Neto et al., 2019; D’Amico-Damião et al., 2020). For the post-emergence management of *A. tenella*, the application of triazine herbicides is recommended at early stages, when the plants show between two and four leaves (Adapar, 2022). In this mode of application, the herbicide acts through contact and undergoes little translocation. Thus, after the nanoencapsulation process, it is believed that there may be gains in translocation due to the interaction of the nanoparticles with plant tissues (Takeshita et al., 2021), promoting greater weed control.

In this context, we aimed to evaluate the post-emergence herbicidal activity of nanoatrazine compared to the commercial formulation in *A. tenella* plants. We also carried out a metanalysis to compare the gain in efficiency provided by nanoencapsulation among the weed species evaluated up to now by our research group.

## 2 Material and methods

### 2.1 Formulations

Nanoatrazine (*nano*ATZ) was synthesized by nanoprecipitation according to Grillo et al. (2012), resulting in nanocapsules of poly(epsilon-caprolactone) (PCL) loaded with atrazine at 1 mg mL^−1^. This methodology consists of preparing two phases: (i) organic phase, obtained by dissolving 100 mg of PCL, 200 mg of myritol, 60 mg of SPAN 60, and 10 mg of atrazine in 30 mL of acetone; and (ii) aqueous phase, contained Tween 80 at 2 mg mL^−1^. To form the nanoparticles, 30 mL of organic phase were added to 30 mL of aqueous phase under magnetic stirring. After 30 min, the volume of the formulation was reduced to 10 mL by evaporation of the solvent. The nanoparticles prepared for this study had 243 ± 5 nm, a polydispersity index of 0.06 ± 0.06, and zeta potential of -30 ± -2 mV. The loading of atrazine by the nanoparticles was 95%. The commercial formulation of atrazine (ATZ) used in the present study was Gesaprim® 500 CG (SC, Syngenta).

### 2.2 Experimental conditions

An initial experiment was conducted in field conditions, in an agricultural area in Londrina, Paraná, Brazil (23°20′24.7″S 51°12′36.6″W). This area was cultivated with second season maize and infested by *A. tenella* plants. Based on the results of Oliveira et al. (2015), the dose of 200 g active ingredient (a. i.) ha^−1^ was chosen to compare the efficiency of *nano*ATZ and ATZ in post-emergence weed control. The experiment also included a control treatment, without the application of any formulation. The experimental plots were 6 m^2^ (with an evaluation area of 3 m^2^), in four replicates. Formulations were applied between 7:00 and 8:00 am at 300 L ha^−1^, using a CO_2_ pressurized backpack sprayer, with a 2-m bar containing four fan nozzles 110.04 spaced 50 cm apart. The treatment was carried out when maize plants showed three to four expanded leaves and *A. tenella* plants were at the four-leaf stage.

The second experiment was conducted in a greenhouse to evaluate the dose-dependent responses of *A. tenella* plants to the atrazine formulations. Seeds of *A. tenella* were collected in the same agricultural area as the initial experiment. They were sown directly in 1-L pots (20 seeds per pot, 1 cm deep). The pots (10.5 cm high, 9.5 cm inferior diameter, 14 cm superior diameter) were filled with soil collected from an herbicide-free portion of the same area. The experiment was carried out in a completely randomized design and organized in a 2 × 4 (atrazine formulations x doses) factorial scheme, constituted for formulations of *nano*ATZ and ATZ, in doses of 200, 500, 1000, and 2000 g a. i. ha^−1^. Some plants were cultivated in pots without the application of any formulation (control). The experiment included five replicates for each treatment and each experimental unit was composed of one pot with five plants with four expanded leaves. The pots were watered every two days, maintaining the soil moisture close to the field capacity. The formulations were applied on a single day (between 7:00 and 8:00 am), spraying 5.1 mL per pot. On the day of application of the treatments, the soil was not watered.

The chemical characteristics of the soil experiments are shown in Table 1 (base saturation was calculated as stated in Equation 1), and the weather conditions prevailing during the experiments are shown in Table 2.

(1) BS (%) = [(K + Ca + Mg)/CEC] x 100

**Table 1:** Chemical characteristics of the soil used in the experiments with *Alternanthera tenella* plants.

pH (CaCl_2_)	OM	P	K	Na	Ca	Mg	SB	CEC	BS
	g dm^−3^	mg dm^−3^	cmol_c_ dm^−3^						%
4.83	28.2	7.63	0.65	0	3.96	1.80	6.41	11.0	58.2

OM: Organic matter; P: Phosphorus; K: Potassium; Na: Sodium; Ca: Calcium; Mg: Magnesium; SB: Sum of bases; CEC: Cation exchange capacity (pH 7.0); BS: Base saturation.

**Table 2:** Weather conditions prevailing during the experiments carried out during 2018 in Londrina, Paraná, Brazil.

		T. Maximum (°C)	T. Minimum (°C)	T. Average (°C)	GSR (MJ m^−2^)	RH (%)
Experiment under field conditions	April	27.6	17,5	22.4	547.6	80.1
	May	25.0	15.0	19.7	462.6	79.1
	June	23.4	14.7	18.7	302.9	84.2
Experiment under greenhouse conditions	September	26.2	15.2	20.6	448.5	81.0
	October	27.2	17.2	22.1	448.0	88.5

T.: Temperature; GSR: Global Solar Radiation accumulated; RH: Relative humidity. *Source*: EMBRAPA, 2019.

### 2.3 Evaluations

The maximum quantum efficiency of PSII (F_v_/F_m_ ratio) of the plants was evaluated using a portable fluorometer (model OS1p, Opti-Sciences, Hudson, USA), at eight, 24, 48, and 96 hours after application (HAA) of the treatments in the field, and at eight, 24, 48, 72, and 96 HAA of the treatments in the greenhouse, following the protocol described by Sousa et al. (2020).

Weed control was assessed 21 days after application of treatments in the field, and seven days after the application of the treatments in the greenhouse. Evaluation was performed visually by assigning a plant damage percentage or death in each experimental unit. A scale from zero to 100% was used, where zero means the absence of any macroscopic symptoms (control plants) and 100% is the death of all plants in the experimental unit.

In the greenhouse experiment, the plants were harvested after this evaluation and their roots washed in running water. The shoot and root materials were immediately weighed on a semi-analytical scale to obtain the fresh mass. After incubation for 72 h at 60 °C, the plant material was weighed again to obtain the dry mass.

### 2.4 Data preparation and statistical analysis

For the field experiment, calculations were carried out with the F_v_/F_m_ data to obtain the percentage of PSII inhibition induced by each treatment compared to control (Equation 2). The data (PSII inhibition and weed control) were transformed in arcsine√x and tested for normality of errors and homogeneity of variances. A one-way analysis of variance (ANOVA) was carried out to compare the effects of the formulations (*p* ≤ 0.05).

In the second experiment, the percentage of PSII inhibition and mass reduction induced by each treatment compared to control were calculated following Eqs. (2) and (3), respectively. The data were transformed in arcsine√x and tested for normality of errors and homogeneity of variances. A two-way ANOVA was carried out to evaluate the effects of the formulations, doses, and their interaction (*p* ≤ 0.05). When significant effects were detected, the means of the formulations were compared by the Tukey test (*p* ≤ 0.05) and the means of doses were submitted to a regression analysis.

All statistical analyses were performed using RStudio statistical software (Version 1.3.1093, RStudio, PBC).

(2) $$\text{PSII}\;\text{inhibition}\;\left(\text{\%}\right)=\frac{Fv/Fm\;Control\;-Fv/Fm\;Treatment}{Fv/Fm\;Control}\;x\;100$$

(3) $$\text{Mass}\;\text{reduction}\;\left(\text{\%}\right)=\frac{Mass\;Control\;-Mass\;Treatment}{Mass\;Control}x\;100$$

### 2.5 Meta-analysis

For meta-analysis, we used data on all weed species for which the herbicidal activity of *nano*ATZ has been studied by our research group: *B. juncea* (Oliveira et al., 2015), *A. viridis* and *B. pilosa* (Sousa et al., 2018), *D. insularis* with two and four leaves expanded (Sousa et al., 2020), *R. raphanistrum* (Takeshita et al., 2021), and *A. tenella* (present study). From these species, *D. insularis* is the only one that showed tolerance to atrazine.

The meta-analysis was performed with PSII inhibition (at 24, 48, and 72 HAA) and shoot dry mass reduction data. Before the analysis, it was necessary to calculate the inhibitory rate (%) from previously published data, using Eqs. (2) and (3). These parameters were chosen because they were available in all published studies. We chose data of the lowest and highest doses utilized in each study (200 and 2000 g a. i. ha^−1^ for all species, except for *D. insularis* in which the lowest tested dose was 1000 g a. i. ha^−1^, given its previously observed tolerance to the herbicide).

The statistical analyses were performed using RStudio statistical software (Version 1.3.1093, RStudio, PBC).

## 3 Results

### 3.1 Herbicidal activity against *Alternanthera tenella*

In both experiments, the photosynthetic evaluations of control plants (without application of any formulation) showed F_v_/F_m_ values close to 0.8 (data not shown), indicating plants with normal photosynthetic activity.

**Figure 1:**
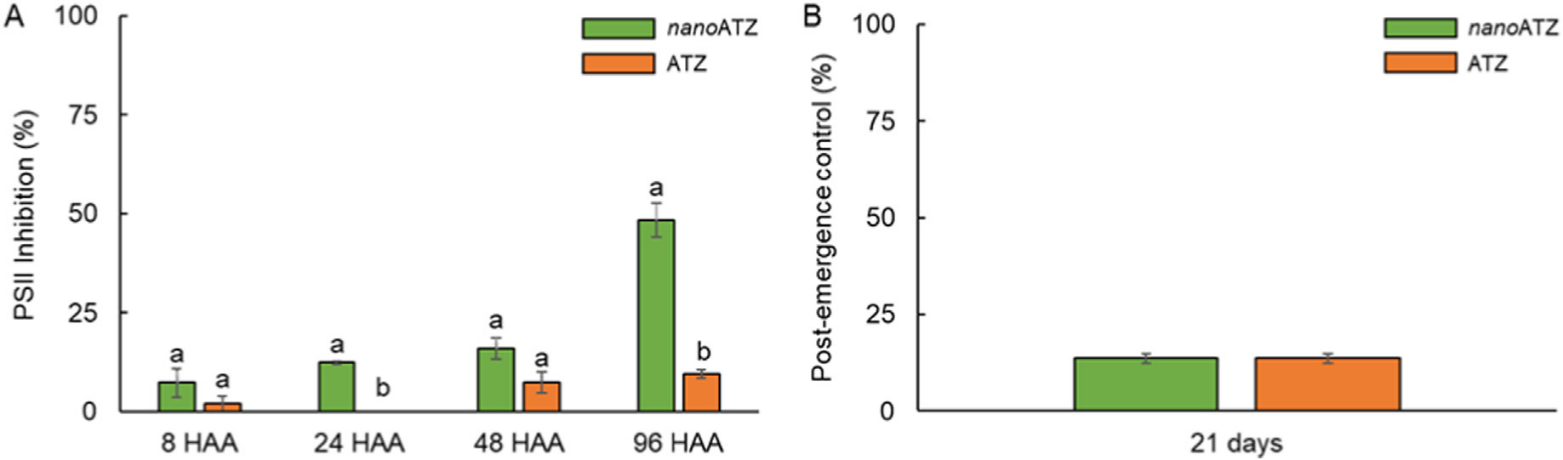
(A) Inhibition of photosystem II (PSII) activity of *Alternanthera tenella* plants at eight, 24, 48, and 96 hours after application (HAA) of nanoatrazine (*nano*ATZ) and commercial atrazine (ATZ) at 200 g a. i. ha^−1^ under field conditions **(**B) Post-emergent control of *Alternanthera tenella* plants provided by the application of nanoatrazine (*nano*ATZ) and commercial atrazine (ATZ) at 200 g a. i. ha^−1^ under field conditions (n = 4).

Figure 1A shows the inhibition of PSII activity of *A. tenella* plants by nanoatrazine (*nano*ATZ) and commercial atrazine (ATZ) in field conditions. Plants treated with *nano*ATZ demonstrated an increment in PSII inhibition, that was 12 and 39% higher than ATZ plants, 24 and 96 hours after application (HAA), respectively. However, weed control did not differ between *nano*ATZ and ATZ treatments (Figure 1B). These results indicate the need for a more detailed evaluation of the response of *A. tenella* plants to *nano*ATZ.

Thus, we carried out a second experiment in a greenhouse using different doses of the herbicide. Table 3 shows the summary of the ANOVA carried out with data from this experiment.

**Table 3:** Summary table of the analysis of variance (ANOVA) for the photosystem II inhibition at eight, 24, 48, 72, and 96 hours after application (HAA), and reduction in shoot fresh-weight (SFR), root fresh-weight (RFR), shoot dry-weight (SDR), and root dry-weight (RDR).

V.F.	8 HAA	24 HAA	48 HAA	72 HAA	96 HAA	SFR	RFR	SDR	RDR
	Fc Value								
Formulation (F)	303.939 ∗∗	49.572 ∗∗	38.802 ∗∗	0.781 ^ns^	0.577 ^ns^	0.311 ^ns^	0.104 ^ns^	0.728 ^ns^	0.203 ^ns^
Dose (D)	20.961 ∗∗	7.076 ∗∗	17.928 ∗∗	9.637 ∗∗	6.107 ∗∗	10.277 ∗∗	1.994 ^ns^	2.122 ^ns^	1,412 ^ns^
F x D	19.798 ∗∗	2.761 ^ns^	1.722 ^ns^	2.581 ^ns^	1.026 ^ns^	0.442 ^ns^	0.466 ^ns^	0.805 ^ns^	1.292 ^ns^
C.V. (%)	6.02	8.84	4.40	4.32	6.51	11.62	5.24	19.37	5.09

V.F.: Variation factor; C.V.: Coefficient of variation.; ∗∗ significant at *p* ≤ 0.01; ∗ significant at *p* ≤ 0.05; ^ns^ not significant.

**Figure 2:**
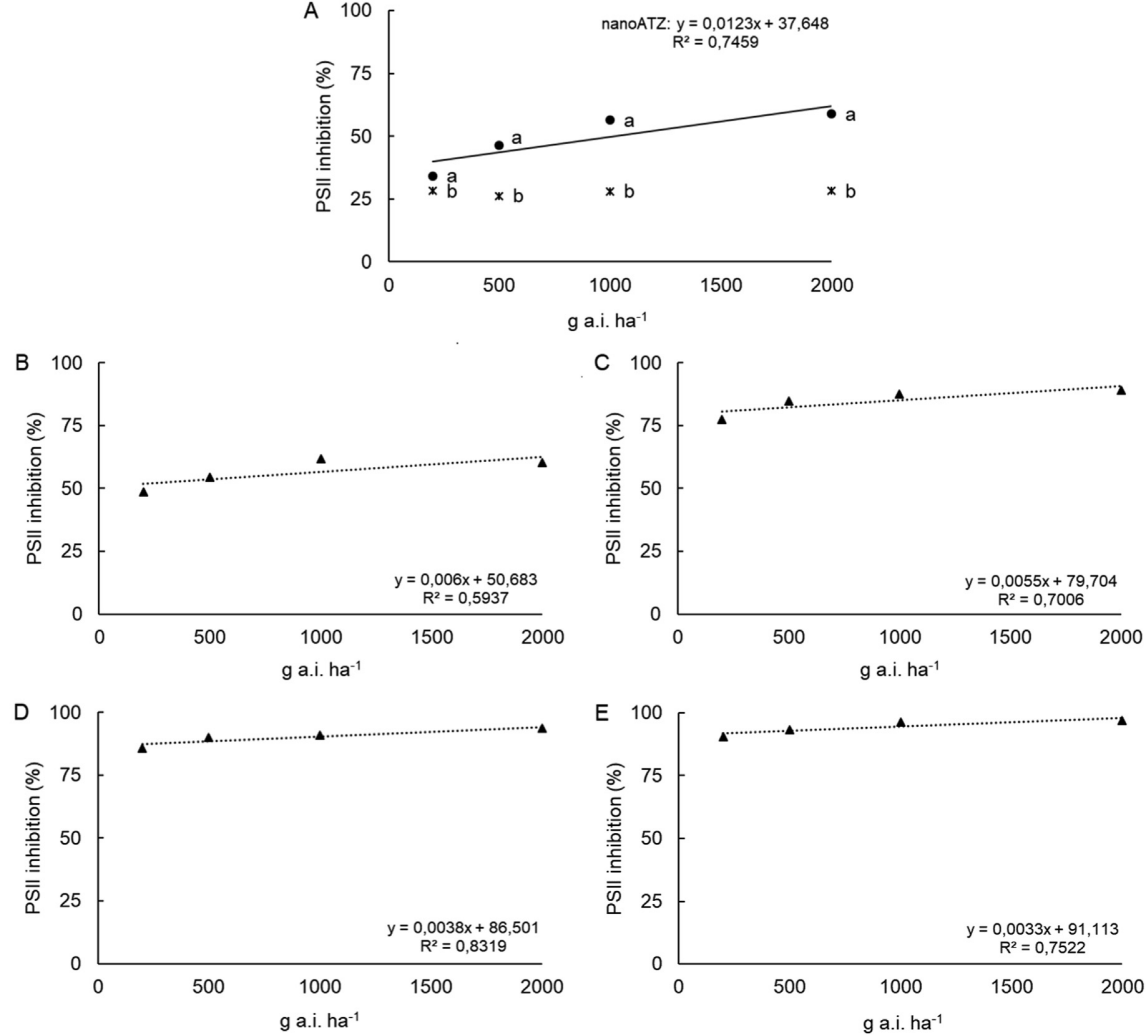
Inhibition of photosystem II (PSII) activity of *Alternanthera tenella* plants at **(**A) eight **(**B) 24 **(**C) 48 **(**D) 72, and **(**E) 96 h after application (HAA) of nanoatrazine (*nano*ATZ) and commercial atrazine (ATZ) at 200, 500, 1000, and 2000 g a. i. ha^−1^ under greenhouse conditions. In **(**A), lower case letters represent a significant difference among ATZ and *nano*ATZ doses by the Tukey test (*p* ≤ 0.05) (n = 5).

In the first evaluation (eight HAA), the interaction between the factors was significant. More expressive inhibitions of PSII activity were observed in plants treated with *nano*ATZ, with means linearly adjusted from 34 to 58%. Meanwhile, ATZ led to inhibition of PSII activity in the range of 30%, not significant for the regression analysis (Figure 2A). Within each dose, inhibition values of PSII activity induced by *nano*ATZ were 6%, 20%, 28%, and 30% higher than inhibition by ATZ at 200, 500, 1000, and 2000 g a. i. ha^−1^, respectively.

From 24 HAA, the interactions between the factors were no more significant and only the independent factors (formulation and dose) were significant (Figure 2B). In this evaluation, atrazine led to percentages of PSII inhibition increasing linearly between 48 and 60%, regardless of the formulation. However, regardless of the dose, the inhibitions of PSII activity were 33% higher in plants treated with *nano*ATZ compared to ATZ.

At 48 HAA, the inhibition of PSII activity by *nano*ATZ was 8.6% higher than ATZ. Regardless of the formulation, the inhibition of PSII activity was linearly adjusted to between 77 and 89% (Figure 2C). At 72 HAA, it was no longer possible to observe differences between the formulations. In this evaluation, the inhibition of PSII activity of *A. tenella* plants varied linearly from 85 to 93% (Figure 2D).

In the final evaluation (96 HAA), regardless of the formulation, atrazine led to high percentages of PSII inhibition (between 90 and 96%). The inhibitions induced by *nano*ATZ and by ATZ did not differ from each other (Figure 2E).

**Figure 3:**
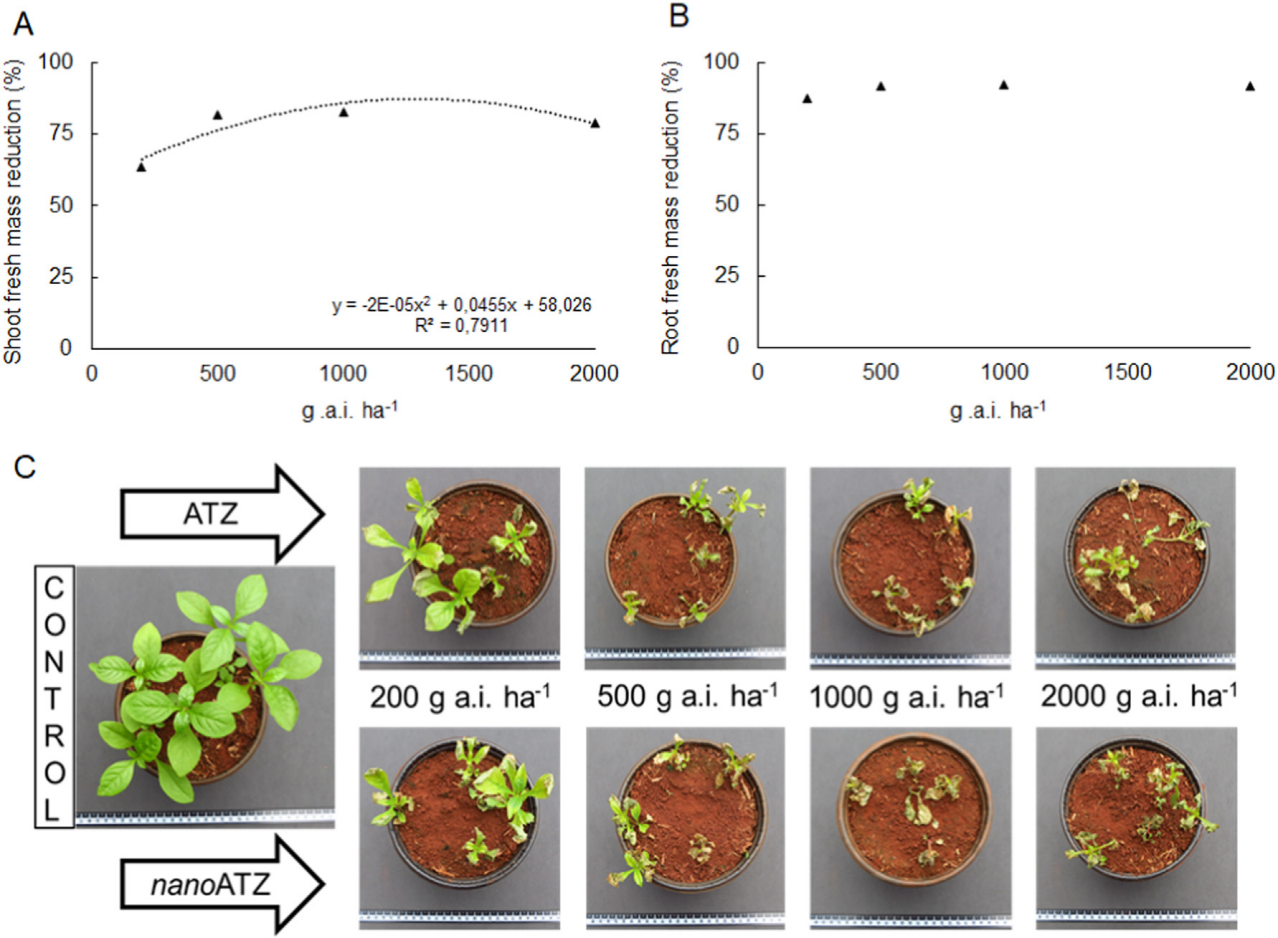
Reduction of **(**A) shoot and **(**B) root fresh mass of *Alternanthera tenella* plants harvested seven days after post-emergence application of nanoatrazine (*nano*ATZ) and commercial atrazine (ATZ) at 200, 500, 1000, and 2000 g a. i. ha^−1^ under greenhouse conditions (n = 5) **(**C) Photographs of representative experimental units of each treatment at seven days after application.

The reduction in shoot fresh mass of *A. tenella* plants was higher for the doses 500, 1000, and 2000 g a. i. ha^−1^ (near to 80%) compared to 200 g a. i. ha^−1^ (63%) (Figure 3A). The reductions in shoot and root dry masses were close to 50% and 90%, respectively, not differing among doses or formulations (data not shown).

**Figure 4:**
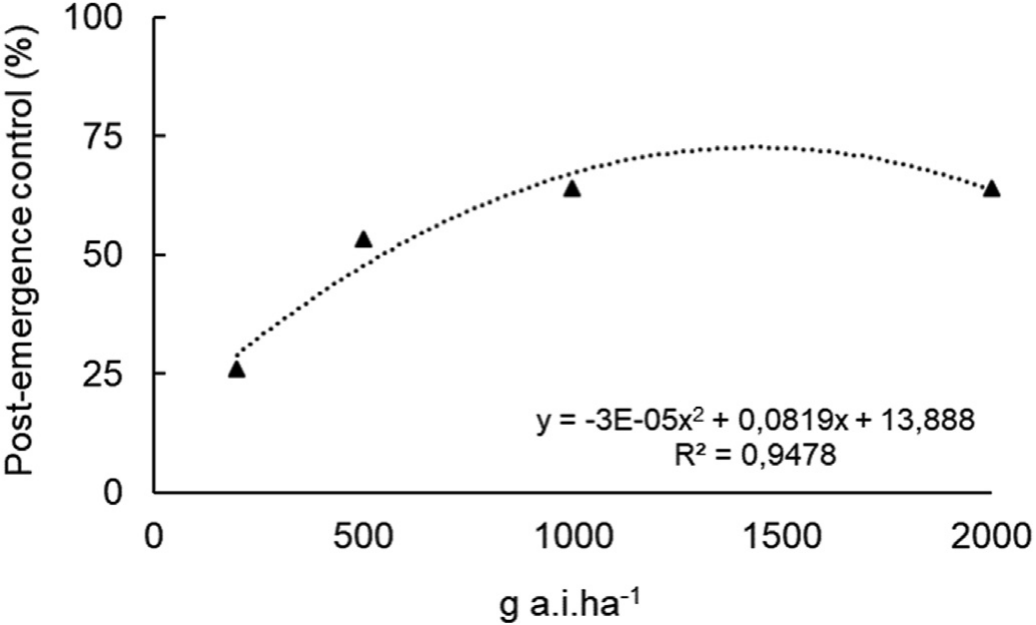
Post-emergent control of *Alternanthera tenella* plants provided by the application of nanoatrazine (*nano*ATZ) and commercial atrazine (ATZ) at 200, 500, 1000, and 2000 g a. i. ha^−1^ under greenhouse conditions (n = 5).

Despite the severe macroscopic symptoms induced by the treatments, regrowth was observed in some experimental units (Figure 3C). This reflected in unsatisfactory weed control (evaluated seven days after the application), as it did not reach the 80% recommended (Figure 4). Regardless of the formulation, the lowest percentage of control of *A. tenella* plants (26%) was induced by 200 g a. i. ha^−1^. For the other doses, means of weed control of 53, 64, and 64% were observed for 500, 1000, and 2000 g a. i. ha^−1^.

### 3.2 Meta-analysis

**Figure 5:**
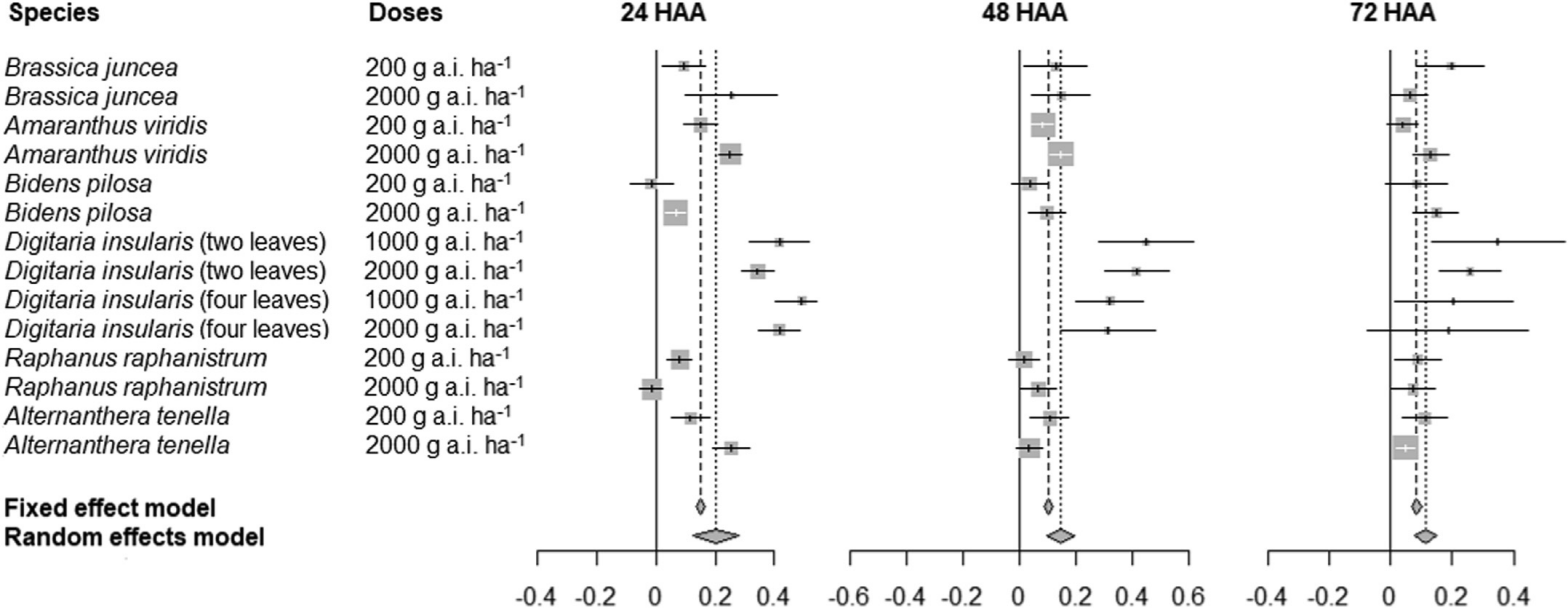
Forest plot summarizing the results of the meta-analysis that evaluated the efficiency of nanoatrazine compared to commercial atrazine in inhibiting photosystem II activity at 24, 48, and 72 h after application (HAA) in six weed species. Horizontal lines (for each species) represent the confidence interval of the data. The size of the OR (*odds ratio*) point of each line is directly proportional to its weight in the analysis. OR points to the left of the central axis indicate that the event is more likely to occur in the control group (higher efficiency of commercial atrazine), while OR points to the right indicate that the event is more likely to occur in the experimental group (higher efficiency of nanoatrazine). When the horizontal line or the OR points touch the central axis (confidence interval containing zero), the difference between formulations is null. Fixed effect model: a result that considers the variability within each study. Random effects model: a result that considers the variability between studies.

The meta-analysis with the results of inhibition of PSII activity from the present study, together with the results from previously published studies are shown as a forest plot graphic (Figure 5). At 24 HAA, *nano*ATZ induced higher inhibitions of PSII activity than ATZ in most cases, as the OR points are represented to the right of the central axis. In the other time-points (48 and 72 HAA), most of the OR points approached the central axis, but all maintained positive values.

In the three time-points, the meta-analysis showed high heterogeneity between species (supplementary material), which is probably related to the different levels of susceptibility or tolerance to atrazine of each weed species. The values of OR points were higher in *D. insularis* (a species that is tolerant to atrazine), which indicates the higher gain in efficiency induced by *nano*ATZ compared to ATZ.

**Figure 6:**
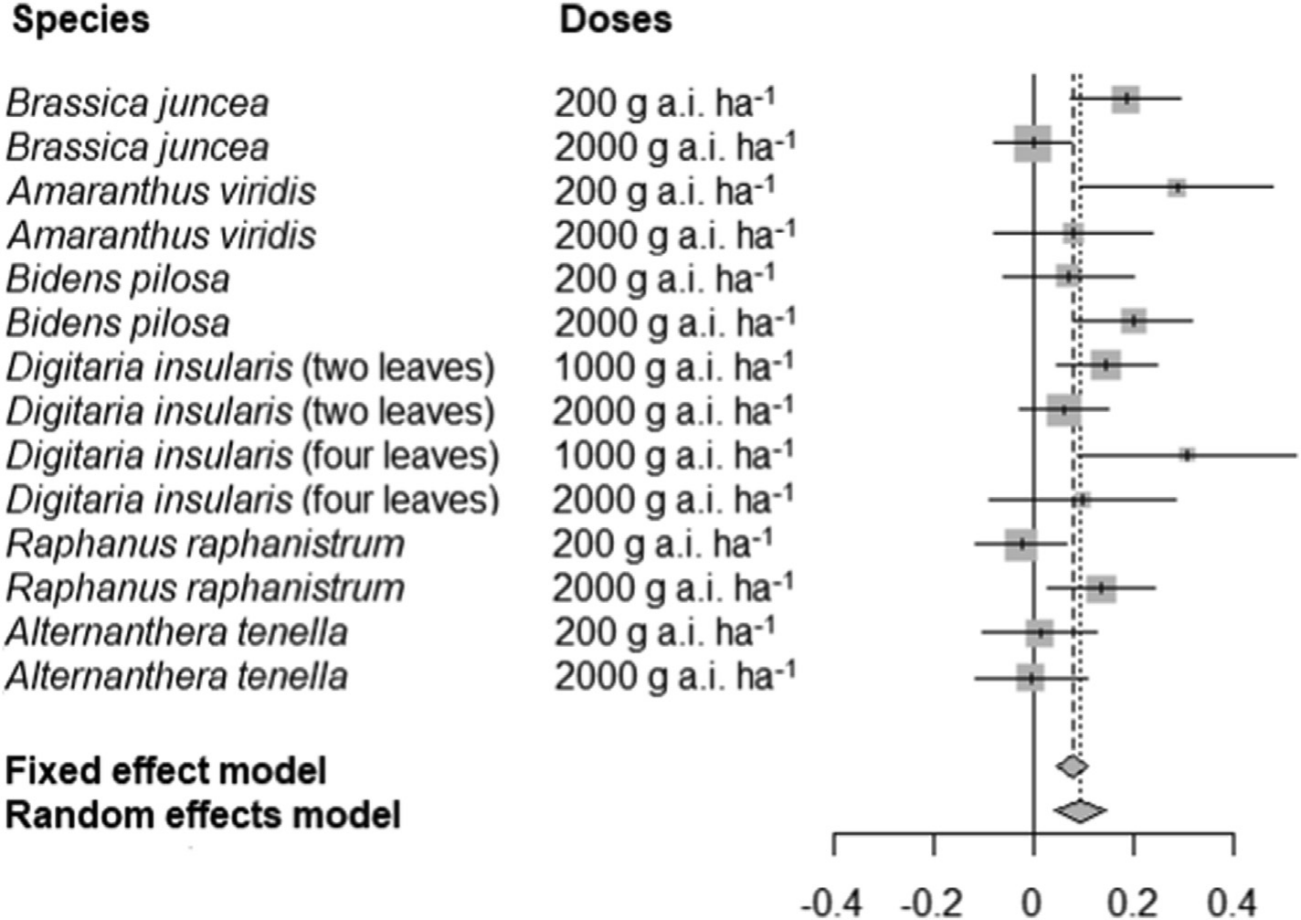
Forest plot summarizing the results of the meta-analysis that evaluated the efficiency of nanoatrazine compared to commercial atrazine in reducing shoot dry mass of six weed species. Horizontal lines (for each species) represent the confidence interval of the data. The size of the OR (*odds ratio*) point of each line is directly proportional to its weight in the analysis. OR points to the left of the central axis indicate that the event is more likely to occur in the control group (higher efficiency of commercial atrazine), while OR points to the right indicate that the event is more likely to occur in the experimental group (higher efficiency of nanoatrazine). When the horizontal line or the OR points touch the central axis (confidence interval containing zero), the difference between formulations is null. Fixed effect model: a result that considers the variability within each study. Random effects model: a result that considers the variability between studies.

Figure 6 presents the forest plot graphic of a meta-analysis with the results of a reduction in shoot dry mass induced by the formulations. Regardless of the dose, the reductions in shoot dry mass of *A. tenella* plants were similar between formulations; therefore, the OR points appear near to the central axis (Figure 5). For the other species, positive values of OR points were obtained, which indicates higher efficiency of *nano*ATZ compared to ATZ (except *B. juncea* at 1000 g a. i. ha^−1^ and *R. raphanistrum* at 1000 g a. i. ha^−1^, that were close to zero).

## 4 Discussion

The application of atrazine to plants hampers the performance of their photosynthetic apparatus and the conversion of light to chemical energy (Dayan et al., 2019). In the current study with *A. tenella* plants, through the evaluations of chlorophyll fluorescence, it was possible to affirm that the atrazine molecules arrived at the action site, since significant reductions in the F_v_/F_m_ ratio were obtained in the treatments with the *nano*ATZ and ATZ formulations.

Higher inhibition of PSII activity by *nano*ATZ compared to ATZ was observed at 24 HAA in the field and until 48 HAA in the greenhouse. Previous studies have demonstrated that nanocapsules without atrazine did not lead to significant phytotoxic effects, which suggested that the enhanced inhibition of PSII activity by *nano*ATZ is caused by the components of the nanoformulation *per se* (Oliveira et al., 2015; Sousa et al., 2018). In contrast, there is evidence that the improved bioactivity of *nano*ATZ is related to greater uptake of the nanoherbicide by natural apertures of leaves, such as stomata and hydathodes (Bombo et al., 2019). In the study of Takeshita et al. (2021), the uptake of *nano*ATZ at 24 HAA was twice the uptake of ATZ in the same period. An altered affinity between the nanocapsules and the leaf surface/plant cell wall that regulates uptake and translocation should also be considered (Fincheira et al., 2020), as well as the facilitated diffusion of the active ingredient into plant tissues, after release by relaxation of the polymeric matrix (Grillo et al., 2012; Pereira et al., 2014).

Despite enhancement in atrazine-induced inhibition of PSII activity by nanoencapsulation, this gain in efficiency was not reflected in differential biomass accumulation and weed control of *A. tenella* plants compared to ATZ. For satisfactory weed control, the active ingredient needs to arrive at its active site in adequate quantities (Dias et al., 2003). However, at the moment of application, the plants had four expanded leaves, and it is possible that some leaf regions and axillar buds were not completely covered by the formulation, thus compromising the herbicidal activity.

Atrazine is a molecule that arrives at its site of action mainly by root uptake and xylem transport, with low mobility via the phloem (Silva et al., 2013). However, in post-emergence applications, the leaves are the main entry routes for the active ingredient into the plants (Thompson and Slife, 1970). Thus, morphoanatomical and physiological particularities of each species can influence the amount of herbicide that is retained on the leaf surface and the amount that is absorbed and translocated (Ferreira et al., 2003; Machado et al., 2008). *A. tenella* leaves are known to have a thick cuticle and wax layer, which act like a physical barrier that hinders the penetration of herbicides (Ferreira et al., 2003). In addition, *A. tenella* leaves have low stomatal density on both faces (Ferreira et al., 2003), which can negatively influence *nano*ATZ uptake (Bombo et al., 2019; Takeshita et al., 2021).

In *B. juncea* plants, Takeshita et al. (2021) reported that *nano*ATZ had enhanced xylem translocation in the treated leaf compared to ATZ, and that atrazine nanoencapsulation led to a minimal increase in leaf-to-leaf phloem translocation. However, some plants have mechanisms of metabolization or compartmentalization of the active ingredient, that can confer selectivity to the herbicides after uptake (Yu and Powles, 2014). Thus, the unsatisfactory post-emergence control of *A. tenella* plants under field and greenhouse conditions (less than 80%), regardless of the formulation type, is possibly related to physical or metabolic barriers and low phloem mobility, that hinders the arrival of atrazine to young organs of *A. tenella* plants (including the “protected” axillary buds), allowing the regrowth of the weed.

Compared to the present study, Takeshita et al. (2021) observed different results in experiments with the application of *nano*ATZ for the post-emergent control of *R. raphanistrum* plants under field and greenhouse conditions. In that study, the increase in PSII inhibition was also reflected in better weed control by *nano*ATZ in both experimental conditions. In *Lactuca sativa* L., similar macroscopic symptoms were also found between nanoatrazine and its non-encapsulated formulation, such as leaf yellowing and necrosis, as well as biomass reduction (Wu et al., 2021). However, the authors indicate that, in the nanoencapsulated form, atrazine led to different effects mainly on nutrient uptake, which were attributed to the altered atrazine release and its increased delivery to plants.

*A meta*-analysis enables better analytic power of a model, improving the chances of evidencing differences between treatments and reinforcing the hypothesis that one treatment has or does not have an effect (Lovatto et al., 2007). Regardless of the variation in herbicidal activity among the different weed species, it was observed that *nano*ATZ is more efficient than ATZ in inhibiting PSII activity for all studied species. When evaluating the reduction in shoot dry mass, *A. tenella* was the only species in which nanoencapsulation did not improve the atrazine effect at any dose. This observation justifies novel experiments verifying the herbicidal activity of *nano*ATZ against other weed species, as well as the investigation of their tolerance mechanisms.

Although the potentiation of the effect of the atrazine promoted by nanoencapsulation was not observed for *A. tenella*, we would still recommend the application of *nano*ATZ. In previous studies, this nanoformulation has already been shown to be more efficient than conventional atrazine against other weed species (Sousa et al., 2018, 2020; Takeshita et al., 2021). Even in the case of *A. tenella*, some benefits might be provided by the use of the nanopesticide, such as lower vulnerability of the active ingredient to environmental factors, decreased deleterious effects towards many non-target species and lower environmental contamination (Grillo et al., 2012; Pereira et al., 2014; Andrade et al., 2019; Takeshita et al., 2021). Moreover, the faster action of *nano*ATZ in compromising the photosynthetic activity of *A. tenella* plants would decrease at some extent their competition with plants of agricultural interest.

## 5 Conclusions

Nanoatrazine showed higher efficiency than commercial atrazine in inhibiting the PSII activity of *A. tenella* until 48 hours after application. However, the mass reduction and weed control did not differ between formulations. The meta-analysis indicated that *A. tenella* showed a different response to *nano*ATZ compared to other species, with a lower gain in efficiency provided by nanoencapsulation.

## Declarations

### Author contribution statement

Bruno Teixeira de Sousa, M. Sc; Anderson do Espírito Santo Pereira, Dr; Leonardo Fernandes Fraceto, Dr; Halley Caixeta de Oliveira, Dr; Giliardi Dalazen, Dr: Conceived and designed the experiments; Performed the experiments; Analyzed and interpreted the data; Contributed reagents, materials, analysis tools or data; Wrote the paper.

### Funding statement

Bruno Teixeira de Sousa was supported by Coordenação de Aperfeiçoamento de Pessoal de Nível Superior - CAPES [Finance Code 001]. This research project is supported by São Paulo Research Foundation (FAPESP) [2017/21004–5], Conselho Nacional de Desenvolvimento Científico e Tecnológico [306583/2017–8 and 311034/2020–9].

### Data availability statement

Data included in article/supp. material/referenced in article.

### Declaration of interests statement

The authors declare no conflict of interest.

### Additional information

No additional information is available for this paper.
